# Diagnostic and Prognostic Value of Circulating Cell-Free DNA for Cholangiocarcinoma

**DOI:** 10.3390/diagnostics11060999

**Published:** 2021-05-30

**Authors:** Preawwalee Wintachai, Jing Quan Lim, Anchalee Techasen, Worachart Lert-itthiporn, Sarinya Kongpetch, Watcharin Loilome, Jarin Chindaprasirt, Attapol Titapun, Nisana Namwat, Narong Khuntikeo, Apinya Jusakul

**Affiliations:** 1 Biomedical Sciences Program, Graduate School, Khon Kaen University, Khon Kaen 40002, Thailand; preawi@kku.ac.th; 2 Cholangiocarcinoma Research Institute, Khon Kaen University, Khon Kaen 40002, Thailand; anchte@kku.ac.th (A.T.); sarinyako@kku.ac.th (S.K.); watclo@kku.ac.th (W.L.); jarich@kku.ac.th (J.C.); attati@kku.ac.th (A.T.); nisana@kku.ac.th (N.N.); knaron@kku.ac.th (N.K.); 3 Lymphoma Genomic Translational Research Laboratory, Division of Medical Oncology, National Cancer Centre and Duke-NUS Medical School, Singapore 169857, Singapore; lim.jing.quan@nccs.com.sg; 4 Centre for Research and Development of Medical Diagnostic Laboratories, Faculty of Associated Medical Sciences, Khon Kaen University, Khon Kaen 40002, Thailand; 5 Department of Biochemistry, Faculty of Medicine, Khon Kaen University, Khon Kaen 40002, Thailand; woracle@kku.ac.th; 6 Department of Pharmacology, Faculty of Medicine, Khon Kaen University, Khon Kaen 40002, Thailand; 7 Department of Internal Medicine, Faculty of Medicine, Khon Kaen University, Khon Kaen University, Khon Kaen 40002, Thailand; 8 Departments of Surgery, Faculty of Medicine, Khon Kaen University, Khon Kaen University, Khon Kaen 40002, Thailand

**Keywords:** bile duct cancer, cell-free DNA, circulating tumor DNA, prognosis, diagnosis, liquid biopsy, sequencing

## Abstract

The analysis of cfDNA has been applied as a liquid biopsy in several malignancies. However, its value in the diagnosis and prognosis of cholangiocarcinoma (CCA) have not been well defined. We aimed to investigate the diagnostic and prognostic values of cfDNA level and tumor-specific mutation in circulating DNA (ctDNA) in CCA. The plasma cfDNA levels from 62 CCA patients, 33 benign biliary disease (BBD) patients and 30 normal controls were quantified by fluorescent assay. Targeted probe-based sequencing of 60 genes was applied for mutation profiling in 10 ctDNA samples and their corresponding treatment-naïve tissues. cfDNA levels in CCA were significantly higher than those in BBD and normal controls. We found that cfDNA levels at 0.2175 and 0.3388 ng/µL significantly discriminated CCA from healthy controls and BBD with 88.7 and 82.3% sensitivity and 96.7 and 57.6% specificity, respectively. cfDNA levels showed superior diagnostic efficacy in detecting CCA compared to CEA and CA19-9. *ARID1A* (30%), *PBRM1* (30%), *MTOR* (30%), and *FGFR3* (30%) mutations were the most common. Using nine frequently mutated genes in the ctDNA samples, the diagnostic accuracy of cfDNA sequencing was 90.8%, with 96.7% average sensitivity and 72.4% specificity. This study supports the use of cfDNA as a diagnosis and prognostic biomarker for CCA.

## 1. Introduction

Cholangiocarcinoma (CCA) is a malignancy that arises from epithelial cells lining the biliary tract [1]. It is the second most common liver cancer and has recently become increasingly prevalent worldwide with a predilection for Southeast Asia. Several risk fac-tors, mainly associated with chronic gallbladder, biliary tract inflammation and liver fluke infection, have been identified [1,2], of which, *Opistorchis viverrini* (Ov) is the dominant liver fluke that is known to be involved in CCA development. It is endemic in Thailand, Vietnam, and Lao PDR and accounted for hotspots of CCA in these regions. Recent studies have also shown that most Thai CCA patients are positive for both anti-Ov antibody and Ov infection [3,4]. These findings point to a link between liver fluke infection and the development of CCA in this area. CCA is often a deadly disease, with very poor prognosis of 7–20% for 5-year overall survival [5,6]. CCA is usually asymptomatic in the early stages and is commonly diagnosed at late stages. Diagnosis of CCA at an early stage could improve long-term patient outcome [7]. To date, histological biopsy remains as the standard tool for diagnosis, but these are limited, due to procedural risks for disease monitoring [8,9]. Currently, serum levels of carcinoembryonic antigen (CEA) and carbohydrate antigen 19-9 (CA19-9) are commonly used to help in the diagnosis and prognosis of CCA, but these are unreliable due to their low sensitivity and specificity, especially for early stages of the disease [10,11]. As such, a minimally invasive procedure and more effective biomarker for CCA are urgently needed.

In recent years, circulating cell-free DNA (cfDNA) and circulating tumor DNA (ctDNA) have been demonstrated to provide prognostic value and disease-monitoring potential in multiple cancers, including biliary tract cancers [12,13]. cfDNA is an extracellular DNA that is thought to be released into the bloodstream by apoptotic and/or necrotic cells [14]. Increased levels of cfDNA in the blood are frequently observed in cancer patients and quantitative changes of cfDNA are correlated with prognosis and tumor burden of the patients. The combined usage of cfDNA with age and alpha-fetoprotein (AFP) was reported to improve the diagnostic performance for HCC [15]. Of note, the diagnostic power of cfDNA was superior to that of AFP in hepatitis C virus-related HCC [16]. Additionally, ctDNA has been shown to carry tumor-specific genetic alterations. As such, tumor-representative ctDNA has been proposed as an adequate alternative to solid tumor biopsies, with the ability to provide diagnostic value, predict responsiveness to treatment and patient survival [17,18]. The concordance between the mutations detected in the ctDNA and their corresponding primary tumors has been shown in biliary tract cancers [18,19,20]. In the ctDNA of CCA patients, therapeutically relevant genomic alterations of the *BRAF*, *ERBB2*, *FGFR2*, and *IDH1* genes were also reported [21]. Moreover, serial analysis of cfDNA in CCA demonstrated polyclonal secondary *FGFR2* mutations that drive acquired resistance to FGFR inhibition [22,23]. These findings highlight the potential advantages of cfDNA analysis in the monitoring and clinical management of patients undergoing targeted therapy and provide useful information to guide the selection of treatment. Although analysis of cfDNA present an attractive diagnostic tool in biliary tract cancers, very limited effort has been made to investigate the diagnostic or prognostic values of cfDNA in comparison to serum protein tumor markers in CCA.

To the best of our knowledge, the diagnostic value of cfDNA compared with current tumor markers, CA19-9 and CEA, has never been investigated in CCA patients. While previous studies emphasize the distinct pattern of genetic alterations in CCA tumors with non-Ov etiology [24,25], here, we subject the ctDNA in CCA from Ov endemic areas and their corresponding treatment-naïve CCA tissues to deep sequencing for genetic profiling. Additionally, we also comparatively quantitate the plasma levels of cfDNA in CCA patients compared to patients with benign biliary disease and healthy individuals as a control. We further compared the diagnostic efficiency of the cfDNA level with serum CA19-9 and CEA.

## 2. Materials and Methods

### 2.1. Patients and Samples

**Figure 1:**
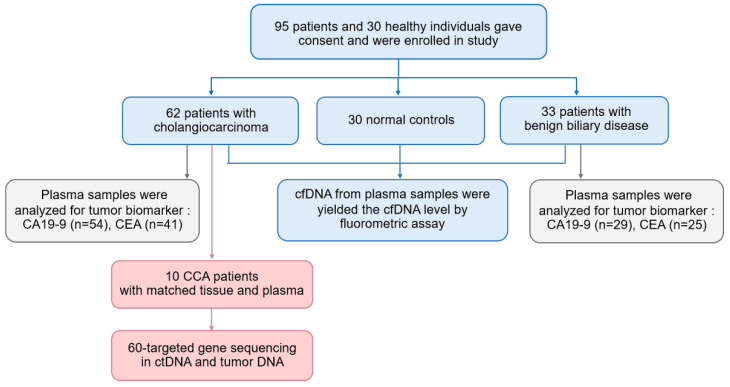
The study design for measuring the diagnostic performance of a plasma cfDNA level and cfDNA-based NGS for CCA patients.

We included patients who had undergone surgery at Srinagarind Hospital and Khon Kaen University between January 2019 and December 2020. Biopsies were performed in all cases and were pathologically diagnosed by a pathologist using the International Collaboration on Cancer Reporting recommendation. The pathological TNM staging system was used according to the 7th AJCC. Blood samples were collected from 62 CCA patients (Supplementary Table S1) and 33 patients with benign conditions including chronic cholecystitis, simple biliary cyst, and biliary cystadenoma, namely, benign biliary disease group (BBD) (Supplementary Table S2). Furthermore, 10 CCA patients (Stage I-II, *n* = 4 and stage III-IV, *n* = 6) were selected for mutational profiling using targeted next-generation sequencing. Matched tissue samples were collected from these 10 patients which included fresh frozen CCA tissues (*n* = 4) and formalin-fixed, paraffin-embedded (FFPE) CCA tissues (*n* = 6). Blood and tissue samples were obtained prior to surgery. Clinical data, levels of CA19-9 and CEA were obtained from the Cholangiocarcinoma Research Institute (CARI). We also included a control group, which comprised 30 healthy blood donors who have no history of any malignancy (Figure 1, Supplementary Table S3). All patients and control individuals were informed and signed the consent form for inclusion in this study. This study has been approved by Human Ethics Committee of Khon Kaen University (HE611556).

### 2.2. Plasma, cfDNA and Genomic DNA Isolation

Blood samples were collected in 10 mL EDTA tubes before surgery. Samples were centrifuged for 10 min at 3500 rpm at 4 °C within 4 h of blood collection. The collected plasma was further centrifuged at 15 min at 8000× *g* at 4 °C to remove additional cellular debris. Plasma was stored at −80 °C until used. cfDNA was extracted from plasma using the QIAamp MinElute ccfDNA Kit (Qiagen) according to the manufacturer’s instructions. Isolated cfDNA was kept at −20°C until required for use. For isolation of genomic DNA (gDNA) from buffy coat, the RBC in buffy coat were lysed using 1× RBC lysis buffer 3 times. gDNA was extracted from buffy coats with the QIAamp DNA Blood Mini Kit (Qiagen) according to the manufacturer’s instructions.

### 2.3. Isolation of Tumor DNA from Fresh and FFPE CCA Tissues

Tumor DNA was extracted from either fresh frozen tissues or FFPE corresponding to the availability of samples. A QIAamp DNA Mini Kit (QIAGEN, Hilden, Germany) was used for DNA isolation from 25 mg of fresh tissue as per the manufacturers’ instructions. For isolation of tumor DNA from FFPE tissue, 8 μm-thick tissue sections were transferred to membrane slides. To estimate the tumor containing area, hematoxylin and eosin stained FFPE tissue slices were identified. The tumor-harboring areas were marked and laser capture microdissection (LCM: MMI CellCut) material was micro-dissected and subjected to a DNA extraction procedure using the QIAamp DNA FFPE tissue kit (QIAGEN, Hilden, Germany) according to the manufacture’s instructions.

### 2.4. Determination of cfDNA Level

Plasma cfDNA levels were determined with a Quant-iT PicoGreen dsDNA Assay Kit (Thermo Fisher Scientific, Vantaa, Finland), according to the manufacturer’s instructions. High range standard curve was prepared using serially diluted Lambda DNA standard (provided by the manufacturer). Fluorescence intensity was measured with a SpectraMax Gemini XPS fluorescent plate reader (Thermo Fisher Scientific Oy)

### 2.5. Customization of Targeted Next-Generation Sequencing Panel

Capture-based probes were designed for 60 genes based on the following criteria; (i) the mutated genes were previously reported with high frequency in CCA [24], and (ii) the mutated genes that were reported in the genomics-driven therapy (TARGET) gene database [26]. The custom targeted enrichment library was designed to capture exons of selected genes with SureDesign’s intuitive design wizards (Agilent). A custom-designed SureSelect bait library captured 60 CCA gene panel including *TP53*, *ARID1A*, *KRAS*, *SMAD4*, *BAP1*, *APC*, *PBRM1*, *ELF3*, *ARID2*, *STK11*, *RNF43*, *SF3B1*, *ACVR2A*, *RASA1*, *BRCA2*, *FBXW7*, *IDH1*, *BRAF*, *RB1*, *PIK3R1*, *PTEN*, *TGFBR2*, *NRAS*, *MAP2K4*, *NCOR1*, *CTNNB1*, *IDH2*, *GNAS*, *KMT2C*, *ATM*, *PIK3CA*, *ERBB2*, *CDKN2A*, *ERBB4*, *FGFR2*, *NF1*, *ROS1*, *BRCA1*, *EGFR*, *ROBO2*, *ATRX*, *GNAQ*, *EPHA2*, *FGFR1*, *HRAS*, *EZH2*, *FGFR3*, *ALK*, *MYC*, *MDM2*, *TERT*, *MAP2K1*, *MAP2K2*, *CDH1*, *CDK4*, *CDK6*, *CDK12*, *MLH1*, *MTOR*, and *FGFR4*.

### 2.6. Library Preparation and Sequencing

Targeted sequencing was performed on cfDNA, matched gDNA and tumor DNA from 10 CCA patients. Sequencing libraries were generated from a total of 40 ng DNA using Agilent SureSelectXT-HS according to the manufacturer’s protocol for each sample. Molecular barcode (MBC) was tagged to DNA fragment for a unique identifier and indexed DNA libraries. The libraries were then sequenced for 150 bp paired-end reads on Illumina NovaSeq 6000 S4 flow cell. The average base coverage depths for ctDNA, and gDNA were 1627× and 1446×, respectively. For tumor DNA, the average base coverage depths on target regions were 2512× and 359× for fresh tumor DNA and FFPE DNA, respectively.

Base-calling and de-multiplexing was performed on the raw sequencing data with bcl2fastq (Illumina, San Diego, CA, USA) software to generate the FASTQ sequencing. FASTQ files were checked and trimmed using fastp software [27]. The preprocessed sequencing reads were aligned to the human genome reference sequence (GRCh37) with BWA MEM [28]. SAMtools was used to sort the alignments and Picard Mark Duplicates routine removed the PCR duplicates [29]. For variant calling, bases with a minimum quality score of 15 were considered by VarScan2.4 tools for somatic variant calling from cfDNA and tumor DNA samples [30]. Functional annotation of somatic variants was applied to the resultant variants using wANNOVAR (Wang Genomics Lab, Philadelphia, PA, USA) [31].

After functional annotation, silent single-nucleotide variants and non-frameshifting insertion/deletion were excluded to focus on variants of potential clinical significance. We then applied additional filters by the following criteria:

(i) Variants supported with reads from both strands, with ≥2 supporting reads, a sequencing depth of ≥200 and a variant allele frequency of ≥0.1 were reported.
(ii) The *p*-value of the candidate somatic mutation to be <0.05.
(iii) Polymorphisms annotated in dbSNP database were discarded from the somatic analysis [32], except polymorphisms which were reported in COSMIC database [33].
(iv) The variants present in more than 30% of patients and absent from the COSMIC cancer mutation database were discarded from the somatic analysis too. The final filtering step was manual examination of variant-supporting alignments with the Integrative Genomics Viewer (IGV) software [34].

### 2.7. Concordance between ctDNA and Tumor DNA Analysis

Mutations in ctDNA were compared to mutations in tumor DNA, and concordance was determined for all mutations across the cohort (mutation-level concordance). Mutated genes that were detected in either ctDNA or tumor DNA per patient were determined for patient-level concordance. Concordance rates and Cohen’s kappa coefficients between mutated genes of tumor DNA and ctDNA were calculated to test an agreement. For the diagnostic performance of the ctDNA sequencing, tissue-based NGS was used as the reference and compared to somatic mutation of ctDNA by gene list. The somatic mutations presented in both matched tumor DNA and ctDNA samples were assigned as true positives. Matched sample pairs without mutations detected in the 60 target genes were assigned as true negatives. The mutations presenting only in ctDNA were indicated as false positives and the mutations presenting only in tissue DNA were indicated as false negatives. Sensitivity, specificity, and rate of concordance ((true positive + true negative)/*n*) were calculated.

### 2.8. Statistical Analysis

SPSS Statistics 23.0 (IBM Corp., Armonk, NY, USA) was used to conduct statistical analyses. All figures were generated by using GraphPad Prism 8.0 (GraphPad Software, Inc., La Jolla, CA, USA). The cfDNA levels were represented as means. For non-parametric statistics, Kruskal−Wallis or Mann−Whitney U test were applied to compare plasma cfDNA levels between clinical features. Dunn’s post hoc test was used for pairwise comparison. The diagnostic performance of cfDNA levels, CA19-9 and CEA were investigated using receiver operating characteristic (ROC) curve analysis, area under the ROC curve (AUC) with 95% CI, and Youden index (YI). Odds ratio was analyzed to predict risks score. The AUC was used to evaluate the predictive ability for the diagnosis. Univariate and multivariate logistic regression analyses were performed to consider the relative contributions of various factors (age, gender) and plasma cfDNA level for the diagnosis. Values of *p* < 0.05 were considered statistically significant. Sensitivity, specificity, positive predictive values (PPV) and negative predictive values (NPV) were determined to evaluate the diagnostic efficacy of cfDNA analysis. Positive detection rates of plasma ctDNA/cfDNA versus tumor biomarkers were calculated as the number of true positives divided by the total number of samples.

## 3. Results

### 3.1. Levels of Plasma cfDNA Increased in Cholangiocarcinoma (CCA) Patients

**Figure 2:**
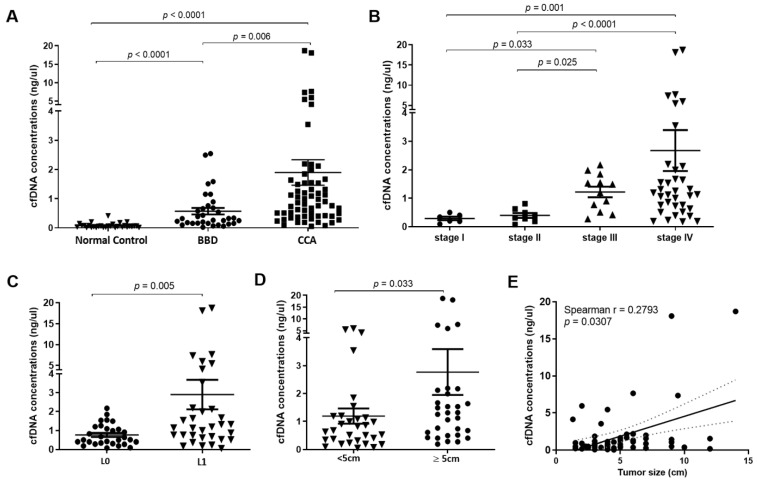
Plasma cell-free DNA (cfDNA) concentration. (**A**) Concentration of plasma cfDNA in CCA was significantly higher than normal control and patients with benign biliary disease (BBD) (*p* < 0.0001, Kruskal−Wallis test). (**B**) Concentration of cfDNA in CCA stage IV was significantly higher than stage I, II and III (*p* < 0.0001, Kruskal−Wallis test). (**C**) CCA patients with lymph node metastasis exhibited a significantly higher cfDNA concentration than CCA patients without lymph node metastasis (*p* = 0.005, Mann−Whitney U Test). (**D**) Concentration of cfDNA in CCA patients with tumor size greater than 5 cm was higher than those with tumor size lower than 5 cm (*p* = 0.033, Mann−Whitney U Test). (**E**) Concentration of cfDNA was positively correlated with tumor size of CCA patients (Spearman r = 0.28; *p* = 0.031). A *p*-value < 0.05 was considered statistically significant.

The clinicopathological characteristics of patients are summarized in Table 1. A total of 95 patients with CCA and BBD, and 30 normal controls were included in this study. This cohort consisted of 76 male and 49 females, and the mean age was 58 years (range 22–81 years). Patients in CCA and BBD groups were older than those in the normal control group (64 ± 8, 60 ± 10, 41 ± 11 years, *p* < 0.001, one-way ANOVA test). There was no significant difference in age between BBD and CCA groups (*p* = 0.137, post hoc tests). We evaluated the levels of plasma cfDNA through a fluorescent assay. The means of cfDNA concentrations were 1.89 ng/µL (range 0.05–18.69 ng/µL), 0.57 ng/µL (range 0.02–2.54 ng/µL) and 0.08 ng/µL (range 0.01–0.41 ng/µL) in patients with CCA, BBD and normal controls, respectively (Figure 2A, Supplementary Tables S1–S3). Multivariate analysis revealed that cfDNA levels and age were independent predictors of CCA (*p* < 0.001, *p* < 0.001, respectively) and BBD (*p* < 0.001, *p* = 0.019, respectively) (Table 2). Overall, the levels of cfDNA in CCA patients were significantly higher than those from normal controls (*p* < 0.0001, *p* < 0.0001 respectively, pairwise Kruskal−Wallis test). Of note, the plasma cfDNA levels from CCA patients were significantly higher than those from BBD patients (*p* = 0.006, pairwise Kruskal−Wallis test).

Moreover, the levels of cfDNA increased according to TMN stage (Figure 2B), the cfDNA levels in CCA patients with stage IV (mean = 2.75 ng/µL) were significantly higher than those from CCA patients with stage I (mean = 0.29 ng/µL) and stage II (mean = 0.39 ng/µL) (*p* = 0.001 and *p* < 0.0001, respectively, pairwise Kruskal−Wallis test). Likewise, levels of cfDNA in CCA patients with stage III (mean = 1.22 ng/µL) were also significantly higher than those from stage I and stage II (*p* = 0.033 and 0.025 respectively, pairwise Kruskal−Wallis test).

We further investigated the cfDNA level in CCA patients with or without lymph node metastasis. The levels of cfDNA from CCA patients with lymph node metastasis (mean = 2.89 ng/µL) were higher than those without lymph node metastasis (mean = 0.76 ng/µL, *p* = 0.005, Mann−Whitney U Test; Figure 2C). Furthermore, the level of cfDNA increased according to tumor size. Levels of cfDNA in CCA patients with a tumor size greater than 5 cm (mean = 2.66 ng/µL) were higher than those with a tumor size lower than 5 cm (mean = 1.19 ng/µL, *p* = 0.033, Mann−Whitney U Test; Figure 2D). Furthermore, we found a weakly positive correlation between cfDNA level and tumor size of CCA patients (Spearman r = 0.28; *p* = 0.031; Figure 2E).

### 3.2. The Diagnostic Efficacy and Predictive Value of Plasma cfDNA Levels

**Figure 3:**
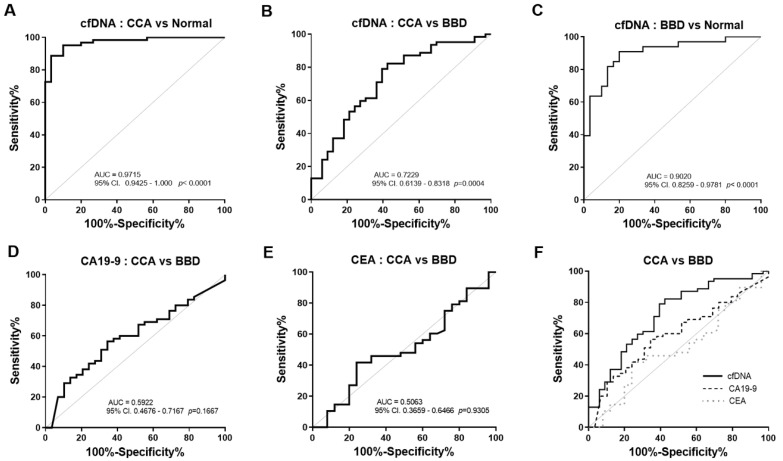
Receiver operating characteristics (ROC) curve analysis for discriminating CCA using plasma cfDNA levels. (**A**) Plasma cfDNA level yielded an AUC of 0.9715 (95% CI: 0.9425–1.000) in discriminating CCA from normal control and (**B**) an AUC of 0.7229 (95%CI: 0.6139–0.8318) in discriminating CCA from BBD. (**C**) cfDNA also showed discriminating ability with an AUC of 0.9020 (95% CI: 0.8259–0.9781) between BBD and normal control. The AUC of CA19-9 (**D**) and CEA (**E**) in discriminating CCA from BBD were 0.5922 (95% CI: 0.4676–0.7167) and 0.5063 (95% CI: 0.3659–0.6466), respectively. (**F**) cfDNA level offered a higher AUC in discriminating CCA from BBD than CA19-9 and CEA. Gray solid line: theoretically perfect performance of a potential biomarker as the reference line. Abbreviations; CCA: cholangiocarcinoma, BBD: benign biliary disease. Area under ROC curve (AUC) are indicated. A *p*-value < 0.05 was considered statistically significant.

We further investigated the diagnostic power of plasma cfDNA level. ROC curves were generated to distinguish CCA patients from normal controls and BBD based on plasma cfDNA level. When compared to normal controls, ROC curve analysis revealed that the cfDNA level showed 88.71% sensitivity and 96.67% specificity to diagnose CCA (AUC = 0.9715, cut-off value 0.2175 ng/µL, *p* < 0.0001, Figure 3A and Table 3). Moreover, the cfDNA level showed 82.26% sensitivity and 57.58% specificity to diagnose CCA compared to BBD (AUC = 0.7229, cut-off value 0.3388 ng/µL, *p* = 0.0004, Figure 3B and Table 3). The cfDNA also showed 90.91% sensitivity and 80% specificity to discriminate BBD from normal control (AUC = 0.9020, cut-off value 0.0897 ng/µL, *p* < 0.0001, Figure 3C and Table 3).

We next determined the predictive value of cfDNA level. In the comparison between CCA and normal controls, multivariate logistic regression analysis (Table 4) revealed that increased cfDNA levels were significantly associated with the diagnosis of CCA (adjusted OR = 227.05, 95% CI (25.93–1988.27), *p* < 0.0001). Additionally, in the comparison between CCA and BBD (adjusted OR values = 7.65, 95% CI (2.72–21.50), *p* < 0.0001). Moreover, OR values revealed that plasma cfDNA level could constantly predict BBD from normal controls (adjusted OR values = 101.46, 95% CI (10.70–961.76), *p* < 0.0001). In contrast, significantly predictive values were not found in CA19-9 (adjusted OR values = 2.18, 95% CI (0.86–5.46), (*p* = 0.099)) and CEA (adjusted OR values = 0.56, 95% CI (0.23–1.37), *p* = 0.204). These results indicated a relatively high diagnostic efficacy of cfDNA level for CCA.

### 3.3. The Diagnostic Performance of cfDNA Levels was Superior to Serum CA19-9 and CEA

CA19-9 and CEA are clinically used as routine tumor markers to diagnose and monitor CCA. However, they have limitations, due to low specificity. We then compared the diagnostic performance of cfDNA level to that of serum CA19-9 and CEA. The cfDNA level showed 82.26% sensitivity and 57.58% specificity to diagnose CCA, compared to BBD (AUC = 0.7229, cut-off value 0.3388 ng/µL, *p* = 0.0004, Figure 3B and Table 3). The ROC curve analysis revealed that the serum CA19-9 level showed 56.36% sensitivity and 65.52% specificity to diagnose CCA compared to BBD (AUC = 0.5922, cut-off value 39.90 U/mL, *p* = 0.1667, Figure 3D and Table 3). The AUC of serum CEA for separating CCA patients from BBD was 0.5063 at the cut-off 2.53 ng/mL with a sensitivity of 41.67% and a specificity of 67% (*p* = 0.9305, Figure 3E and Table 3). Of note, the AUC of the cfDNA level was higher than those of serum CA19-9 and CEA in discriminating CCA from BBD (Figure 3F).

**Figure 4:**
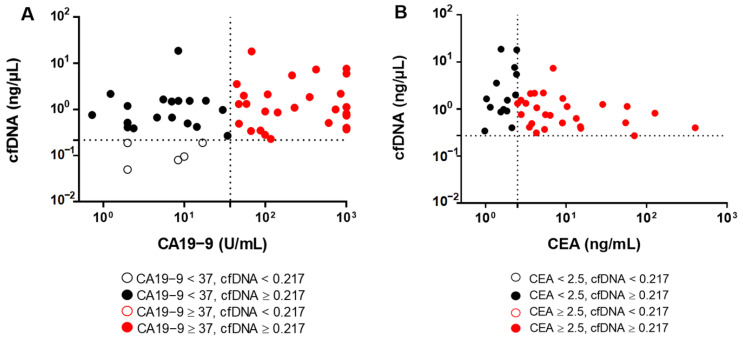
Diagnostic accuracy of cfDNA in comparison with CA19-9 and CEA. The scatter plots show (**A**) the distribution of CA19-9 level vs. cfDNA level and (**B**) the distribution of CEA levels vs. cfDNA level in CCA patients. The optimal cut-off levels of cfDNA level, CA19-9 and CEA are 0.217 ng/µL, 37 U/mL and 2.5 ng/mL, respectively.

The diagnostic accuracy of cfDNA level was compared to serum CA19-9 and CEA (Figure 4). A total of 49/54 (90.74%) of CCA patients were diagnosed using cfDNA level (cut-off level = 0.2175, Figure 4A). Of note, there were 24/54 (44.44%) CCA patients who had levels of CA19-9 lower than the normal range (<37 U/mL). Importantly, there were 19/24 (79.17%) CCA patients with low levels of CA19-9 who were correctly diagnosed using cfDNA, (cut-off value 0.2175 ng/µL). Likewise, there were 14/41 (34.15%) of CCA patients who had CEA levels lower than normal range (<2.5 ng/mL) but they (14/14) were precisely diagnosed as CCA when using cfDNA level ≥ 0.2175 ng/µL as cutoff (Figure 4B). These results suggest that the level of plasma cfDNA is a potential biomarker for the diagnosis of CCA.

### 3.4. Profiling of Somatic Mutation in ctDNA and Diagnostic Sensitivity, Specificity and Accuracy for ctDNA Sequencing

**Figure 5:**
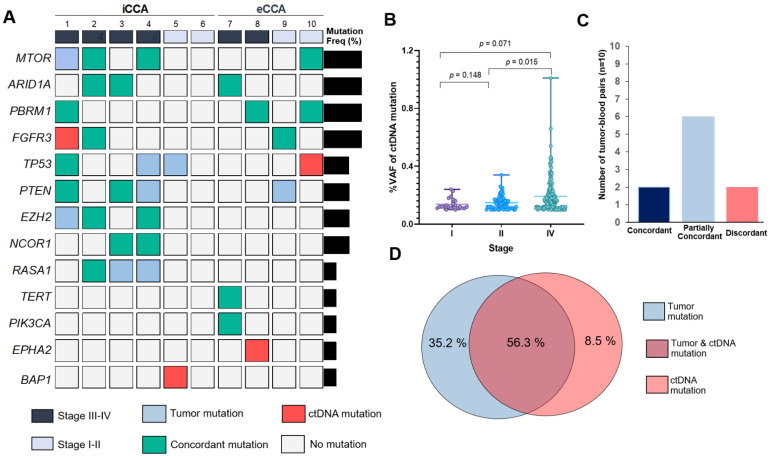
Mutational concordance across 10 CCA patients. (**A**) Onco-print chart showing the characteristic (staging and subtype) and mutation occurrence in ctDNA compared to matched tumor. Only mutated genes (*n* = 13) detected in ctDNA are shown. Bar graph shows the proportions of patients with mutated genes (Right). (**B**) Comparison of variant allele frequency (VAF) percentage in ctDNA stratified according to staging (stage I (*n* = 1), II (*n* = 3) and IV (*n* = 6)). (**C**) Numbers of matched tumor DNA and ctDNA samples in each patient-level concordance category. (**D**) The overlapping of mutations between cfDNA and tissue sequencing tests. iCCA; intrahepatic CCA, eCCA; extrahepatic CCA, Freq%: mutation frequency in patient, ctDNA: circulating tumor DNA.

As mutations found in plasma ctDNA can also provide useful information for targeted therapies, we investigated if plasma ctDNA mutations could determine the treatment selection in CCA. Deep targeted sequencing in 60 genes was used to detect somatic nonsynonymous mutations and small insertions/deletions which presented in ctDNA. At least one characterized somatic mutation was detected in 90% of patients (9/10) for ctDNA mean of somatic mutations per patient: 2.6 (range 0–5)] and 100% (10/10) for tissue-DNA (mean of somatic mutations per patient: 4.5 (range 1–10)). Among 60 target genes, there were 13 genes observed in ctDNA, including *MTOR*, *ARID1A*, *PBRM1*, *FGFR3*, *TP53*, *PTEN*, *EZH2*, *NCOR1*, *RASA1*, *TERT*, *PIK3CA*, *EPHA2* and *BAP1* (Figure 5A, Supplementary Table S4). The most frequently mutated genes in ctDNA were *MTOR* (30%), *ARID1A* (30%), *PBRM1* (30%) and *FGFR3* (30%). Of note, we found that the mean of VAF in ctDNA of CCA patients increased corresponding to tumor staging. The mean of VAF in ctDNA was higher in CCA patients with stage IV (mean: 0.19 ± 0.11), than II (mean: 0.15 ± 0.05), and I (mean: 0.14 ± 0.04) (*p* = 0.071, 0.015, respectively; pairwise Kruskal−Wallis test; Figure 5B).

Seventy-one somatic mutations were reported from the union of all tumors and ctDNA tests across the entire cohort of 10 patients (mean: 7.1 mutations per patient; Supplementary Table S4). The mutational overlapping between ctDNA and tumor tissue sequencing were identified as mutation-level concordance of 56.34% (40/71). There were 35.21% (25/71) of mutations which were found in tumor DNA but not ctDNA. Of note, 8.5% (6/71) of total mutations were observed in only ctDNA but not tumor DNA (Figure 5D). Patients were grouped into three categories, based on the two sets of mutational reports: concordant, partially concordant, and discordant. Concordant samples were those with all detected mutations that were found in both tissue-based and ctDNA-based sequencing tests. Partial concordance was allowed if at least one mutation, but not all mutations, was concordant between tumor DNA and ctDNA. Discordance occurred if no mutation was concordant. Mutational reports from two patients were completely concordant (20%), six were partially concordant (60%) and two were discordant (20%) (Figure 5C).

To assess the efficacy of ctDNA-based testing, we performed gene-level sensitivity and specificity analyses of the ctDNA test for nine recurrent mutated genes (*FGFR3*, *MTOR*, *PTEN*, *PBRM1*, *ARID1A*, *EZH2*, *TP53*, *NCOR1*, and *RASA1*). The tumor tissue–NGS based test was used as the reference. Across the nine genes, the average sensitivity was 96.88% (range 85.71–100%), specificity was 73.08% (range 33.33–100%), and average diagnostic accuracy was 90% (range 70–100%, Table 5). ctDNA sequencing could appropriately predict with the diagnostic probability of 90.48% PPV and 89.86% NPV. Interestingly, the ctDNA based NGS of these genes revealed a substantial level of agreement with tissue-based NGS (Cohen’s κ = 0.742, *p* < 0.001).

### 3.5. Plasma ctDNA Detection Versus Tumor Biomarkers

**Figure 6:**
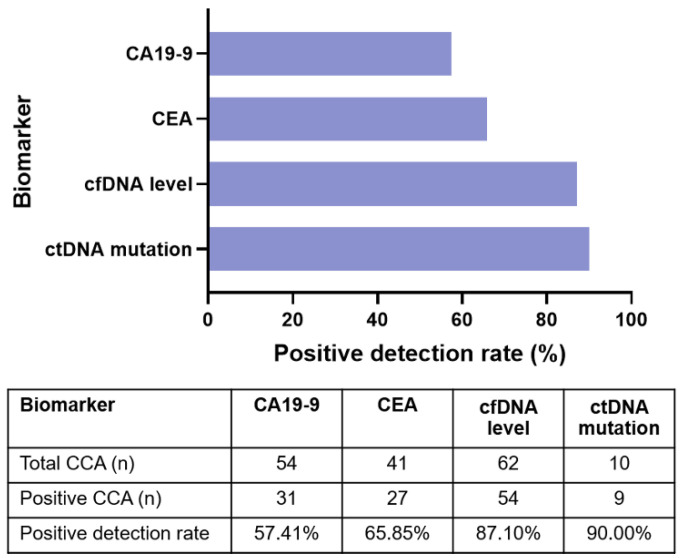
Positive detection rates of plasma cfDNA level and ctDNA sequencing compared to biomarkers.

To evaluate the diagnostic efficacy of plasma ctDNA and comparisons with tumor biomarkers: CA19-9 and CEA, 62 plasma CCA samples were tested for one or more of the biomarkers. Overall, 54/62 (87.10%) blood samples were positive for detection of cfDNA level, 9/10 (90%) were positive for mutations in ctDNA, 31/54 (57.40%) were positive for CA19-9, and 27/41 (65.85%) were positive for CEA (Figure 6). Hence, the level of cfDNA and ctDNA mutations had a higher detection rate and higher PPV compared to CA19-9 and CEA.

## 4. Discussion

Patients with CCA are usually met with poor prognoses and significantly high morbidity rates, due to the limited options for early detection and treatment. Around 90% of patients would die within the first year of being diagnosed [9]. Surgical resection of CCA tumors is the treatment of choice, but most cases are inoperable. Despite the availability of many diagnostic tools, CCA diagnosis remains difficult [35]. In clinical practice, CA19-9 and CEA are the most frequently used blood-based tumor markers [10]. However, the lack of sensitivity and specificity has been a major problem in the use of most serum tumor markers for the diagnosis of cancers. In recent years, cfDNA analysis has received growing attention because of its applications as a surrogate marker for multiple indications in cancer, including diagnosis, prognosis, and monitoring [36]. Additionally, the combination of cfDNA markers with other blood-based tumor markers has recently shown improvement in diagnostic accuracy [37,38]. Moreover, sequencing of ctDNA has the potential to assess the genetic profile with a minimally invasive procedure [21]. Thus, the potential for use of ctDNA in the diagnosis and management of CCA is of interest. However, investigations into the use of ctDNA in CCA have previously been hampered by the rarity of the disease.

Although analysis of cfDNA and ctDNA has been shown to be an attractive diagnostic and prognostic tool in malignancies, including biliary tract cancers [15,20,21,39,40], very few studies have evaluated and compared the diagnostic efficiency of cfDNA analysis with current tumor markers used in clinical practice. In the present study, we assessed the diagnostic and prognostic value of cfDNA level in CCA, particularly to compare with benign biliary diseases using a simple, rapid, and sensitive fluorescence dye-based spectrophotometry as well as investigating the diagnostic value of somatic mutations in ctDNA of CCA patients. We highlight that cfDNA analysis could play an important role in distinguishing benign biliary diseases and CCA and may serve as a noninvasive marker for CCA diagnosis.

We found that the plasma cfDNA levels in CCA were significantly higher than those in benign biliary diseases and normal controls. The mean level of cfDNA in CCA was about 24-fold higher than in the healthy control group and about 3-fold higher than that of the benign biliary disease group. Consistent with previous reports [15,41], the cfDNA concentrations detected in our study corresponded to stage, tumor size and lymph node metastasis, suggesting that cfDNA levels correlate with disease severity and progression of CCA in patients. cfDNA is considered to arrive in circulation by predominately apoptosis of hematopoietic cells and other nucleated cells in a healthy individual. In cancer patients, it is believed to result from the lysis or active release of circulating tumor cells and original cancer necrosis [14]. It should be noted that patients with benign biliary diseases had higher cfDNA compared to the healthy control group, however, they had significantly lower levels of cfDNA when compared to CCA patients. Growing evidence suggested that the elevated levels of cfDNA were not only specific to cancer patients, but also patients with nonmalignant diseases, i.e., autoimmune disorders, myocardial infarction, or pulmonary thromboembolism. Thus, an increased level of cfDNA in plasma/serum is not specific for a defined disease [42,43,44]. It is possible that benign diseases share the same mechanisms with cancer in increasing the cell proliferation that affect the release of cfDNA. It is also conceivable that cfDNA is the result of inflammation caused by benign hyperplasia [45].

We demonstrated cfDNA as a marker to discriminate CCA patients from both benign biliary diseases and the normal healthy control groups. The detection of the plasma cfDNA level achieved a high AUC value (0.972), sensitivity (88.71%) and specificity (96.67%) to discriminate CCA from normal controls. The challenge of CCA diagnosis is to reliably distinguish CCA from benign biliary disease which exhibits the proximate pathogenesis of disease. Interestingly, we found a sensitivity and specificity of 82.26% and 57.58 % to discriminate CCA from benign biliary disease. Current tumor biomarkers including CA19-9 and CEA often show conflicting results and have low specificity. Of note, the diagnostic efficacy of cfDNA level was superior to serum CA19-9 and CEA in the present cohort. Consistent with our findings, the cfDNA was found to be significantly lower in cholecystitis controls and healthy subjects, compared to the gall bladder cancers [46], suggesting that cfDNA quantitative analysis could play an important role in distinguishing benign biliary disease and certain malignancies.

Plasma ctDNA offers a better understanding of the specific disease condition, as the ctDNA originates from tumors and contains mutations only present in tumor cell DNA. Considering the data presented in previous studies that revealed the number of clinically actionable genetic variants in CCA [24,25], the targeted approach chosen may be clinically valuable for the diagnosis of suspicious findings and estimation of prognosis. As CCA genetic variations differ radically by etiology, we demonstrated, for the first time to our knowledge, preliminary investigated somatic mutations of ctDNA in CCA, particularly in endemic areas of liver fluke infection. We designed a gene sequencing panel to identify somatic mutations in mutated genes that were reported in liver fluke associated CCA and mutated genes relevant for genomics-driven therapy. A total of 13 somatic mutated genes were identified in plasma ctDNA, including *ARID1A*, *PBRM1*, *MTOR*, *FGFR3*, *TP53*, *PTEN*, *NCOR1*, *EPHA2*, *PIK3CA*, *TERT*, *RASA1*, *EZH2* and *BAP1*. Mutations in *ARID1A*, *BAP1*, *PBRM1*, and *TP53* were previously reported to be associated with poor prognosis for patients with cancer diseases [47,48,49,50]. *ARID1A* and *PBRM1* encode a subunit of the SWI/SNF complex, and were mostly mutated in CCA, including association with tumor progression [48,49]. Mutations in *ARID1A* and *PBRM1* may also be the target of therapeutic drugs by targeting residual SWI/SNF activity [51]. The loss of chromatin remodeling subunits could impact the response to immune checkpoint therapy such as anti-PD-1/PD-L1 therapy [52]. Of note, one of the patients (C001) had a potentially actionable mutation in the loss of PBRM1 function (p.I279Yfs*3, COSM392156) which was detected in ctDNA, which could be of benefit for immune checkpoint inhibitor treatments in this patient.

*EPHA2*, encodes a transmembrane of the tyrosine kinase family, involving invasion and migration in different types of tumors for instance in CCA [53]. *EPHA2* was found to be frequently mutated in intrahepatic CCA [54]. Patient C008 contained an actionable mutation in *EPHA2* (p.R762H, COSM3782397), suggesting that this patient might benefit from receptor tyrosine kinase inhibitors in metastatic cancer. Additionally, mutations in *MTOR* genes were found in ctDNA. Wu, et al. (2019) suggested that the first-generation (Rapalogs) and second-generation of mTOR-inhibitors might have an anti-tumor effect in CCA [55]. We also found mutation in the PI3K pathway including *PIK3CA* and *PTEN*. *PIK3CA* mutations which are frequently found in CCA and regarded as candidates for targeted therapies in cancers, especially biliary tract cancer [56]. *PTEN* promotes chromosome stability and DNA repair. Loss of PTEN function is associated with cancer progression [57]. The therapeutic drugs of PI3K inhibitors and AKT inhibitors were additional targeted therapies for targeted mutant PIK3CA and/or PTEN patients [58].

Our study has shown that ctDNA-based NGS of a gene panel is feasible and accurately detects tumor-derived mutations in CCA. We achieved a performance of tumor-derived detection of ctDNA-based NGS for a gene panel that is comparable to tissue biopsy with 96.88% sensitivity, 73.08% specificity along with 90% diagnostic accuracy across nine frequently mutated genes in ctDNA of this cohort. ctDNA-based NGS provided a substantial level of agreement with tissue-based NGS, reflecting the high level of concordance between the two platforms. A previous study by Mody et al. performed a ctDNA sequencing on 138 samples of biliary tract cancer, particularly intrahepatic CCA. They found at least one genomic alteration in 89% of cases [21]. A total of 21% of patients were identified as having actionable alterations in *BRAF*, *ERBB2*, *FGFR2*, and *IDH1*. Additionally, ctDNA mutations compared to tissue mutations had a concordance of 74% in all patients and 92% in the intrahepatic CCA cohort [20]. Recently, Okamura et al. identified mutations in *TP53* (38%), *KRAS* (28%), and *PIK3CA* (14%) for ctDNA in biliary tract cancers and demonstrated that 76% of biliary tract cancer had at least one characterized alteration in ctDNA. Overall concordance between ctDNA and tissue-DNA was 68% to 90% for *TP53*, *KRAS* and *PIK3CA* genes [39]. Of note, there were 8% of total mutations observed in only ctDNA but not tissue-DNA. However, there were no other primary tumors observed in these cases. Similar to our findings, Ettrich et al. reported that 8% of mutations found in ctDNA were not seen in the respective CCA tumor sample [20]. Discrepancies between the two tests may be due to the intertumor and intratumor genetic heterogeneity as only segments of the whole tumors were used for sequencing analysis [18,59]. Similarly, some mutations were found in tumor but not ctDNA. These mutations may have been present in circulation but at levels insufficient for accurate detection. A challenge in ctDNA mutation monitoring lies in the detection threshold where in vivo levels may be below detectable limits. Interestingly, we observed that 50% (4/8) of total cases with partially concordant and discordant mutations were early-stage patients (stage I−II). Indeed, ctDNA is detectable in some patients with early-stage cancers [12,60], but assay sensitivity in early-stage cancer has remained a challenge. In most early-stage cancers, the amount of ctDNA is very low which may be reflective of low tumor burden and insufficient to detect mutant fragments of ctDNA releasing in circulation [12,60]. A disadvantage of targeted sequencing of ctDNA is that this method cannot determine if the mutations originated from primary or metastatic tumors; however, because ctDNA levels correlate with tumor burden, an increase in ctDNA may be indicative of disease recurrence or progression.

In addition, the VAF of ctDNA might correlate with tumor burden [61]. We observed that the VAF in ctDNA of CCA patients was increased and related to tumor staging. Consistent with our finding, the VAF of ctDNA showed a correlation with progression-free survival and tumor load in CCA, suggesting that ctDNA quantity may be correlated to clinical tumor load [20,61,62]. Furthermore, five patients with ctDNA mutations had normal CA19-9 levels, suggesting that ctDNA sequencing could provide both tumor burden and mutational information in selected patients who, for whatever reason, do not secrete antigenic tumor markers.

Taken collectively, we provide a rationale of cfDNA/ctDNA analysis that could serve as a biomarker in combination with current blood-based biomarkers to improve diagnostic efficacy. These preliminary findings pave the way for precision oncology approaches in CCA and introduce cfDNA into clinical routine. Our study does have some limitations, including limited sample size, and the fact that patients are derived from a single institution. Further investigations are needed to validate our results in a larger cohort. Additional study is warranted to exploit its full potential.

## 5. Conclusions

The results from our study have shown that ctDNA can be used as a feasible biomarker for CCA. Herein, we highlighted two uses of cfDNA analysis. The first was assessing the efficacy of cfDNA level for CCA diagnosis and prognosis. We showed that cfDNA levels increase in CCA patients when compared to patients with chronic biliary diseases and healthy controls. Levels of cfDNA have been shown to increase in CCA patients with increased tumor progression. The diagnostic efficacy of cfDNA levels is superior to CA19-9 and CEA in CCA diagnosis. The second was examining whether ctDNA sequencing harbors the potential to improve the clinical management of CCA. Mutations detected in ctDNA are relatively representative of the corresponding tumor tissue. It is important to emphasize, however, that the data analyses were performed on a limited sample size, hence, preliminary conclusions can only be advanced. Nonetheless, the study lays a sound foundation from which further investigations on a bigger cohort of representative samples can be conducted.

## Figures and Tables

**Table 1:** Clinico-pathological features of patients and normal controls.

Variables	CCA	BBD	Normal Controls	*p*-Value
**Gender (*n*)**	62	33	30	0.141
male	43	18	15	
female	19	15	15	
**Age *(n)***	62	33	30	<0.001 *
range	51–81	34–79	22–61	
mean ± SD (years)	64 ± 8	60 ± 10	41 ± 11	
**WBC count *(n)***	60	31	-	0.463
range	4.1–52.5	4.3–12.5	-	
mean ± SD (× 10^3^/µL)	8.7 ± 6.5	7.8 ± 2.7	-	
**Neutrophils *(n)***	60	31	-	0.152
range	39.0–85.6	33.6–89.9	-	
mean ± SD (%)	61.9 ± 10.9	58.1 ± 12.9	-	
**Hemoglobin *(n)***	60	31	-	0.796
range	7.7–15.8	7.2–15.5	-	
mean ± SD (g/dL)	12.1 ± 1.6	12.2 ± 1.9	-	
**cfDNA levels *(n)***	62	33	30	0.002 *
range	0.05–18.69	0.02–2.54	0.01–0.41	
mean ± SD (ng/mL)	1.89 ± 3.43	0.57 ± 0.64	0.08 ± 0.09	
**CA19**-**9 levels *(n)***	54	29	-	0.086
range	0.73–1000	2–1000	-	
mean ± SD (U/mL)	271.4 ± 392.1	139.4 ± 290.7	-	
**CEA levels *(n)***	41	25	-	0.361
range	1.0–402.0	0.3–1341	-	
mean ± SD (ng/mL)	22.0 ± 65.4	62.0 ± 266.8	-	
**CCA**			***n* (%)**	
Primary tumor location				
Intrahepatic CCA			31 (50%)	
Extrahepatic CCA			27 (43.5%)	
Intrahepatic and Extrahepatic CCA			4 (6.5%)	
Lymph node metastatic				
Yes			33 (53.2%)	
No			29 (46.8%)	
Tumor size				
<5 cm			31 (50.9%)	
≥5 cm			30 (49.1%)	
Tumor stage				
stage I			6 (9.6%)	
stage II			8 (12.9%)	
stage III			12 (18.8%)	
stage IV			36 (58.1%)	

Chi-Square test was used to determine a significant relationship of nominal (categorical) variables; Gender. Independent T test or one-way ANOVA test were applied to compare the clinical features. The symbol (*) indicates *p*-value < 0.05 that was considered statistically significant.

**Table 2:** Multivariate analysis of cfDNA, age and sex of patients and normal control groups.

Variables	Odds Ratio	*p*-Value	Confidence Interval (95%)	
			Lower	Upper
CCA vs. normal				
cfDNA	227.86	<0.001	26.73	1942.52
age	5.32	<0.001	2.08	13.57
sex	2.26	0.074	0.92	5.55
BBD vs. normal				
cfDNA	32.86	<0.001	7.65	141.13
age	3.45	0.019	1.23	9.74
sex	1.20	0.718	0.45	3.23
CCA vs. BBD				
cfDNA	6.29	<0.001	2.44	16.26
age	2.33	0.123	0.80	6.83
sex	1.96	0.177	0.74	5.20

**Table 3:** The performance of cfDNA, CA19-9, CEA for CCA diagnosis, based on the best cut-off derived from ROC analysis and Youden index.

Group Comparisons	Biomarkers	Cut-Off	AUC (95% CI)	YI	SN (%)	SP (%)	LR	*p*-Value
Normal vs. CCA	cfDNA level (ng/µL)	>0.2175	0.9715 (0.943–1.000)	0.85	88.71	96.67	26.61	<0.0001
Normal vs. BBD	cfDNA level (ng/µL)	>0.0897	0.9020 (0.826–0.978)	0.71	90.91	80.00	4.55	<0.0001
BBD vs. CCA	cfDNA level (ng/µL)	>0.3388	0.7229 (0.614–0.832)	0.40	82.26	57.58	1.94	0.0004
BBD vs. CCA	CA19-9 (U/mL)	>39.90	0.5922 (0.468–0.717)	0.22	56.36	65.52	1.64	0.1667
BBD vs. CCA	CEA (ng/mL)	>2.53	0.5063 (0.366–0.647)	0.18	41.67	67.00	1.74	0.9305

Abbreviations; AUC: area under the ROC curve, YI: Youden index, SN: sensitivity, SP: specificity, LR: likelihood ratio CCA: cholangiocarcinoma, BBD: benign biliary disease. A *p*-value < 0.05 was considered statistically significant.

**Table 4:** The predictive risk of CCA and BBD relative to normal control group by using level of cfDNA, CA19-9 and CEA.

Comparative Diagnosis	Biomarkers	Crude		Adjusted	
		OR (95% CI)	*p*-Value	OR * (95% CI)	*p*-Value
Normal vs. CCA	cfDNA < 0.2175 vs. ≥ 0.2175 ng/µL	227.86 (26.73–1942.52)	<0.0001	227.05 (25.93–1988.27)	<0.0001
Normal vs. BBD	cfDNA < 0.0897 vs. ≥ 0.0897 ng/µL	32.86 (7.65–141.13)	<0.0001	101.46 (10.70–961.76)	<0.0001
BBD vs. CCA	cfDNA < 0.3388 vs. ≥ 0.3388 ng/µL	6.29 (2.44–16.26)	<0.0001	7.649 (2.72–21.50)	<0.0001
BBD vs. CCA	CA19-9 < 39.90 vs. ≥ 39.90 U/mL	2.30 (0.94–5.62)	0.068	2.18 (0.86–5.46)	0.099
BBD vs. CCA	CEA < 2.53 vs. ≥ 2.53 ng/mL	0.61 (0.26–1.42)	0.251	0.56 (0.23–1.37)	0.204

* Odds ratio adjusted for age and sex statistical analysis. Abbreviations; OR: odds ratio, CI: confidence interval, CCA: cholangiocarcinoma, BBD: benign biliary disease. A *p*-value < 0.05 was considered statistically significant.

**Table 5:** The performance of ctDNA-based sequencing analysis for CCA diagnosis.

Gene	ctDNA Mutations	Tumor Mutations		Sensitivity (%)	Specificity (%)	PPV (%)	NPV (%)	False Positive (%)	False Negative (%)	Diagnostic Accuracy (%)
		+	−							
*ARID1A*	+	3	0	100.00	100.00	100.00	100.00	0.00	0.00	100.00
	−	0	7							
*PBRM1*	+	3	0	100.00	100.00	100.00	100.00	0.00	0.00	100.00
	−	0	7							
*NCOR1*	+	2	0	100.00	100.00	100.00	100.00	0.00	0.00	100.00
	−	0	8							
*MTOR*	+	3	0	100.00	75.00	100.00	85.71	0.00	14.29	90.00
	−	1	6							
*EZH2*	+	2	0	100.00	66.67	100.00	87.50	0.00	12.50	90.00
	−	1	7							
*PTEN*	+	2	0	100.00	66.67	100.00	87.50	0.00	12.50	90.00
	−	1	7							
*FGFR3*	+	2	1	87.50	100.00	66.67	100.00	33.33	0.00	90.00
	−	0	7							
*RASA1*	+	1	0	100.00	33.33	100.00	77.78	0.00	22.22	80.00
	−	2	7							
*TP53*	+	1	1	85.71	33.33	50.00	75.00	50.00	25.00	70.00
	−	2	6							
Total	+	19	2	96.88	73.08	90.48	89.86	9.52	10.14	90.00
	−	7	62							

Abbreviations; PPV: positive predictive value, NPV: negative predictive value.

## Data Availability

The targeted sequencing data done in this article have been deposited at the NCBI’s Sequence Read Archive (SRA; https://www.ncbi.nlm.nih.gov/sra, accessed on 30 April 2021) under BioProject ID: PRJNA726346. Deposited date 30 April 2021.

## Supplementary Materials

The following are available online at https://www.mdpi.com/article/10.3390/diagnostics11060999/s1, Table S1: The clinico-pathological data and characteristics of CCA patients; Table S2: The clinico-pathological data and characteristics of BBD patients; Table S3: The characteristics of normal controls; Table S4: Details of the detectable genetic variants in matched plasma ctDNA and tumor tissue from patients with CCA.

## Abbreviations

AUC	Area under the ROC curve
CA 19-9	Carbohydrate antigen 19-9
CCA	Cholangiocarcinoma
cfDNA	Cell-free DNA
CI	Confidence interval
BBD	benign biliary disease
ctDNA	Circulating tumor DNA
LR	Likelihood ratio
NGS	Next-generation sequencing
NPV	Negative predictive value
OR	Odds ratios
PPV	Positive predictive value
ROC	Receiver operating characteristic curve
SD	Standard deviation
SN	Sensitivity
SP	Specificity
VAF	Variant allele frequency
VCF	Variant call format
YI	Youden index
